# Anti-Inflammatory Activity of Cyanobacteria Pigment Extracts: Physiological Free Radical Scavenging and Modulation of iNOS and LOX Activity

**DOI:** 10.3390/md22030131

**Published:** 2024-03-12

**Authors:** Lécia Rodrigues, Janaína Morone, Guilherme Scotta Hentschke, Vitor Vasconcelos, Graciliana Lopes

**Affiliations:** 1 CIIMAR—Interdisciplinary Centre of Marine and Environmental Research, Terminal de Cruzeiros do Porto de Leixões, Avenida General Norton de Matos s/n, 4450-208 Matosinhos, Portugal; lecia.beatriz@hotmail.com (L.R.); janabavini@ciimar.up.pt (J.M.); guilherme.scotta@gmail.com (G.S.H.); vmvascon@fc.up.pt (V.V.); 2 FCUP—Faculty of Sciences, University of Porto, Rua do Campo Alegre s/n, 4169-007 Porto, Portugal

**Keywords:** carotenoids, phycobiliproteins, RAW 264.7 cells, superoxide anion radical, nitric oxide, citrulline

## Abstract

Cyanobacteria are among the oldest organisms colonizing Earth. Their great biodiversity and ability to biosynthesize secondary metabolites through a variety of routes makes them attractive resources for biotechnological applications and drug discovery. In this pioneer study, four filamentous cyanobacteria (*Cephalothrix lacustris* LEGE 15493, *Leptolyngbya boryana* LEGE 15486, *Nodosilinea nodulosa* LEGE 06104 and *Leptothoe* sp. LEGE 11479) were explored for their anti-inflammatory potential in cell and cell-free in vitro bioassays, involving different inflammatory mediators and enzymes. Extracts of different polarities were sequentially prepared and chemically characterized for their content of phycobiliproteins (PBPs) and carotenoids. HPLC-PDA analysis of the acetone extracts revealed β-carotene to be the dominant carotenoid (18.4–44.3 mg/g) and zeaxanthin as the dominant xanthophyll (52.7–192.9 mg/g), with *Leptothoe* sp. LEGE 11479 and *Nodosilinea nodulosa* LEGE 06104, respectively, being the richest strains. The PBP profile was in accordance with the color presented by the aqueous extracts, with *Leptolyngbya boryana* LEGE 15486 being the richest in phycocyanin (204.5 μg/mg) and *Leptothoe* sp. LEGE 11479 the richest in phycoerythrin (78.5 μg/mg). Aqueous extracts were more effective in superoxide anion radical scavenging, while acetone ones were more effective in scavenging nitric oxide radical (^●^NO) and in inhibiting lipoxygenase. Acetone extracts also reduced ^●^NO production in lipopolysaccharide-stimulated RAW 264.7 macrophages, with the mechanistic study suggesting a downregulation of the inducible nitric oxide synthase expression. *Nodosilinea nodulosa* LEGE 06104 and *Leptothoe* sp. LEGE 11479 acetone extracts presented the lowest IC_50_ values for the mentioned assays, pointing them out as promising resources for the development of new multi-target anti-inflammatory therapies.

## 1. Introduction

Inflammatory processes have been recognized as pathophysiological hallmarks in several age-related diseases, with particular relevance in the major chronic diseases among developed populations, including cardiovascular diseases, type 2 diabetes mellitus, asthma, neurodegenerative disorders, and many types of cancer [1]. As part of the normal physiologic response during healing, inflammation provides practical benefits to reinstall tissue homeostasis after injury. However, when prolonged, inflammatory response can become damaging and destructive, leading to lifelong debilitation, accompanied by loss of tissue function and organ failure [2]. Multi-level cell and molecular mechanisms underly this dynamic network, with enzymes, growth factors, cytokines, leukotrienes, prostaglandins and thromboxanes participating in the regulated signaling pathway that characterizes the complex inflammation scenario. Immune cell activation can be triggered by protein-associated molecular patterns derived from pathogens, as well as by danger-associated molecular patterns (DAMP), triggered by a wide range of host-derived endogenous signals, which are recognized by pattern-recognition receptors, of which the tool-like receptors (TLR) are dominant. Cyclooxygenase (COX) and 5-lipoxygenase (5-LOX) are among the DAMP-producing enzymes, synthesizing biolipid molecules through the metabolism of arachidonic acid (AA), such as prostanoids (prostaglandins, prostacyclins and thromboxanes, produced by the action of COX-1 and COX-2) and leukotrienes (hydroxyeicosatetraenoids and lipoxins, produced by the action of 5-, 12-, and 15-LOX) [1]. Together with prostanoids and leukotrienes, reactive species of oxygen (ROS) and nitrogen (RNS) can also act as signal transduction molecules to induce a pro-inflammatory state. Nitric oxide (^•^NO), an RNS with signaling properties, has been a target of intensive research since its discovery. This diffusible messenger is a product of the nitric oxide synthase (NOS) family of enzymes, with its most significant production as the inducible isoform (iNOS), and participates in the regulation of numerous cellular physiological and pathophysiological functions [3]. Among others, NO is known for the regulation of inflammatory transcription factors in both prokaryotic and eukaryotic cells [4].

Given the large variety of mediators involved in the complex inflammatory network, it comes as no surprise that targeting individual mediators is unlikely to produce very effective therapies, reinforcing the shift of the “one drug, one target” paradigm to a multitarget approach as a promising clinical strategy for the management of inflammatory-associated diseases. In this sense, the biological targets and mechanisms of action of natural products have been subjects of increasing focus in the scientific community [5]. Among the wide variety of natural resources, it is noteworthy that cyanobacteria are at the forefront of scientific research. Among other features, these ancient prokaryotic microorganism with cosmopolitan occurrence are endowed with great biodiversity, which is reflected in a wide spectrum of secondary metabolites biosynthesized through a variety of routes and, consequently, having a wide range of biological activities [6]. Carotenoids and phycobiliproteins (PBPs) appear particularly interesting, with some reports demonstrating their potential to modulate different mediators involved in the inflammatory process [7]. For instance, previous reports on the anti-inflammatory activity of isolated carotenoids showed that these compounds can suppress ^•^NO production by lipopolysaccharide (LPS)-stimulated macrophages (RAW 264.7), downregulate the expression of inducible nitric oxide synthase (iNOS) [8] and COX-2, and reduce the release of tumor necrosis factor-α (TNF-α), interleukin (IL)-1β and IL-6 [9]. Furthermore, both carotenoids and PBPs have been recognized for their antioxidant activity, which is a real asset since, through radical scavenging, these compounds can reduce the exacerbation of inflammation, reducing tissue damage and accelerating repair [10]. Thus, it seems clear that pigment-rich extracts from cyanobacteria are worthy of further exploitation, given their multi-target potential to overcome inflammation-associated diseases.

In this work, pigment-rich extracts of four filamentous cyanobacteria strains, *Cephalothrix lacustris* LEGE 15493, *Leptolyngbya boryana* LEGE 15486, *Nodosilinea nodulosa* LEGE 06104 and *Leptothoe* sp. LEGE 11479, were obtained through a sequential extraction process, with their carotenoid profiles being reported herein for the first time. The extracts of different polarities were explored for their anti-inflammatory potential through different cell and cell-free in vitro bioassays, comprising important cells, enzymes and physiological free radicals involved in the inflammatory process. To the best of our knowledge, this is the first report on the anti-inflammatory potential of the mentioned cyanobacteria strains, as well as on the exploitation of their mechanism of action.

## 2. Results and Discussion

### 2.1. Identification of the Strains

**Figure 1:**
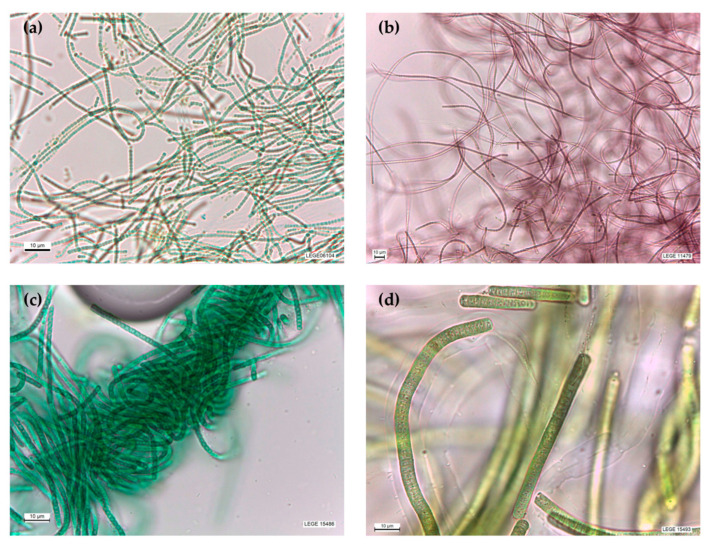
Selected cyanobacteria strains: *Nodosilinea nodulosa* LEGE 06104 (**a**), *Leptothoe* sp. LEGE 11479 (**b**), *Leptolyngbya boryana* LEGE 15486 (**c**) and *Cephalothrix lacustris* LEGE 15493 (**d**). (Photographs kindly provided by the Blue Biotechnology and Ecotoxicology Culture Collection, LEGE-CC, of CIIMAR).

The morphological (Figure 1) and phylogenetic analyses (Figure S1) are in agreement and reveal that the strains LEGE 06104, 14479, 15486 and 15493 are within the circumscription of *Nodosilinea*, *Leptothoe*, *Leptolyngbya* and *Cephalothix*, respectively. The strain LEGE 06104 (Figure 1a) presents greenish constricted trichomes, barrel-shaped cells, and thin colorless sheaths, and is phylogenetically closely related to the reference strain *Nodosilinea nodulosa* PCC7104. The strain LEGE 14479 (Figure 1b) presents reddish slightly constricted trichomes, isodiametric cells, and thin colorless sheaths, and is phylogenetically closely related to the reference strain *Leptothoe sithoniana* TAUMAC0915. The strain LEGE 15486 (Figure 1c) presents greenish unconstricted trichomes, cylindrical cells, and thin colorless sheaths, and is phylogenetically closely related to the reference strain *Leptolyngbya boryana* CCALA 1076. The strain LEGE 15493 (Figure 1d) presents greenish uncostricted trichomes, cells wider than longer, and thin colorless sheaths, and is phylogenetically related to the reference strain *Cephalothrix lacustris* CCIBt 3261 (Figure 1 and Figure S1).

### 2.2. Chemical Characterization

The cyanobacteria extracts were chemically characterized according to their main components, resulting from the sequential extraction process with solvents of different polarities.

#### 2.2.1. Carotenoid and Chlorophylls

**Figure 2:**
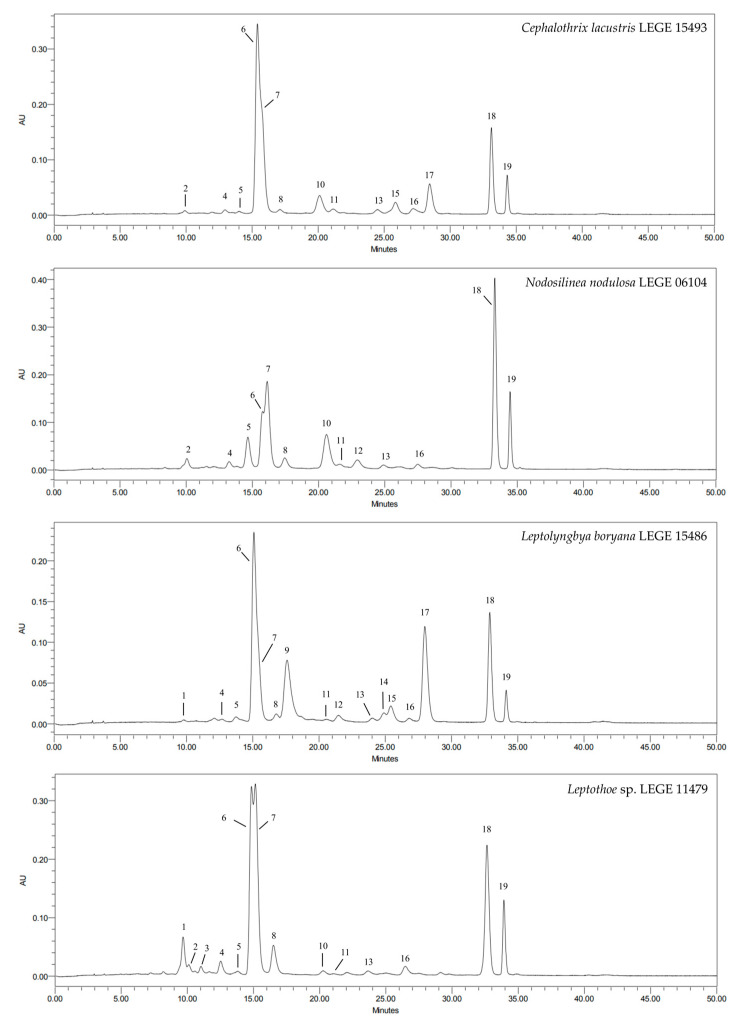
Carotenoid and chlorophyll profiles of acetonic extracts of the cyanobacteria *Cephalothrix lacustris* LEGE 15493, *Nodosilinea nodulosa* LEGE 06104, *Leptolyngbya boryana* LEGE 15486 and *Leptothoe* sp. LEGE 11479. HPLC-PDA recorded at 450 nm. Chlorophyll-*a* derivates (**1**, **2**, **3**, **8**), lutein (**4**), unidentified carotenoids (**5**, **9**, **10**, **11**, **12**, **14**, **16**, **17**), chlorophyll-*a* (**6**), zeaxanthin (**7**), β-cryptoxanthin (**13**), echinenone (**15**), β-carotene (**18**) and β-carotene derivative (**19**).

The carotenoid and chlorophyll profile of the acetone extracts was analyzed with HPLC-PDA, resulting in a good chromatographic resolution, as illustrated in Figure 2.

The identified compounds comprised four xanthophylls: lutein (**4**), zeaxanthin (**7**), β-cryptoxanthin (**13**) and echinenone (**15**); one carotene: β-carotene (**18**); and chlorophyll-*a* (**6**). Furthermore, chromatographic analysis demonstrated the existence of nine compounds with similar UV–VIS spectra to those of the identified carotenoids, but differing in their retention times when compared to the standards, thus being tentatively defined as unidentified carotenoids (**5**, **9**, **10**, **11**, **12**, **14**, **16**, **17**). The same happened with compounds 1, 2, 3 and 8, which displayed spectra with the typical maxima of chlorophyll-*a* (430 and 664 nm), but had different retention times, thus being tentatively classified as chlorophyll-*a* derivates.

The total carotenoid concentration ranged between 128.66 and 260.63 mg/g of dry extract (Table 1). *Leptothoe* sp. LEGE 11479 showed the highest carotenoid content, closely followed by *Nodosilinea nodulosa* LEGE 06104 (260.63 and 228.19 mg/g, respectively). *Cephalothrix lacustris* LEGE 15493 and *Leptolyngbya boryana* LEGE 15486 presented the lowest carotenoid amounts (153.98 and 128.66 mg/g, respectively) (*p* < 0.05). Regarding chlorophylls, their total concentration ranged between 8.23 and 21.48 mg/g of dry extract, with *Leptothoe* sp. LEGE 11479 also being the richest species (21.48 mg/g, *p* < 0.05) (Table 1).

In a more detailed analysis, all strains contained lutein (**4**), chlorophyll-*a* (**6**), zeaxanthin (**7**), β-cryptoxanthin (**13**), and β-carotene (**18**). On the other hand, echinenone (**15**) was only identified in two strains: *Cephalothrix lacustris* LEGE 15493 and *Leptolyngbya boryana* LEGE 15486, *Cephalothrix lacustris* LEGE 15493 being the one displaying the highest amount of this xanthophyll (1.78 mg/g) (*p* < 0.05). Similar to echinenone, chlorophyll-*a* (**6**) stood out for also being in higher quantity in *Cephalothrix lacustris* LEGE 15493 (17.46 mg/g), however with no significant differences when compared to *Leptothoe* sp. LEGE 11479 (*p* > 0.05). Zeaxanthin (**5**) was identified in all strains, with the highest concentration observed in *Leptothoe* sp. LEGE 11479 (192.91 mg/g). It was also possible to verify that lutein (**4**) and β-cryptoxanthin (**13**) also appeared in larger quantities in *Leptothoe* sp. LEGE 11479 (Table 1) (*p* < 0.05). Concerning β-carotene (**18**), this compound was dominant in *Nodosilinea nodulosa* LEGE 06104, however with no significant differences relative to *Leptothoe* sp. LEGE 11479.

Several studies have demonstrated that a state of chronic low-grade inflammation plays an important role in a host of additional chronic diseases and disorders [11]. Carotenoids, with antioxidant and anti-inflammatory functions, may improve these conditions by contributing to a resolution of the inflammatory state. Nevertheless, the profiling and exploitation of the anti-inflammatory potential of pigment-rich cyanobacteria extracts are still scarce. Previous studies have reported β-carotene in different genera of cyanobacteria, such as *Synechocystis*, *Phormidium*, *Cuspidothrix*, *Anabaena*, *Nostoc*, *Aphanothece*, *Cyanobium* and *Leptothoe* [12,13], demonstrating the broad spectrum of cyanobacterial genera producing this compound and, consequently, the importance of these natural resources for biotechnological applications. β-Carotene has pro-vitamin A properties, having great relevance in human health, namely in the prevention of several diseases related to oxidative stress [14]. Together with β-carotene, zeaxanthin is one of the most prevalent carotenoids in cyanobacteria, recognized for its antioxidant capacity, and associated with cancer-preventive properties, improvement in skin health, and maintenance of normal and accurate visual function in humans [15]. Hashtroudi and co-workers [16] reported that the levels of zeaxanthin ranged between 13.3 and 46.4 µg/g of dry weight (DW) in *Anabaena* strains analyzed, which was significantly lower than the values obtained by us (Table 1). The same happened for β-carotene, where the content found by the authors varied between 5.220 and 7.137 mg/g of DW. On the other hand, lycopene was the most representative carotenoid reported by the authors, and was absent in the strains analyzed by us. Besides belonging to a different cyanobacteria species, which constitutes the main reason for the differences observed in the chemical profile, it was extracted via a procedure employing cold methanol, with the polarity of the solvent also influencing the extraction of different compounds.

Studies by Morone et al. [13] and Lopes et al. [17] on the carotenoid profiles of different strains of the genus *Nodosilinea* and *Cyanobium*, showed that these genera contained species with the highest contents of β-carotene. Similar to our study, the strain *Nodosilinea nodulosa* LEGE 06104 had the highest β-carotene content; however, the amount quantified herein (44.315 mg/g) was significantly higher than that obtained by Morone and co-workers (0.041 mg/g). The main reason for this difference is the solvent used for carotenoid extraction; the authors used ethanol 70%, for which these low polar compounds have limited affinity, thus being extracted in minor amounts. Regarding *Leptothoe* sp. LEGE 11479, a previous study with acetone extracts undertaken by our research group [12] reported zeaxanthin and β-carotene to be the major compounds (with 39.9 and 47.9 mg/g of DW, respectively). While the β-carotene content found by the authors was similar to that reported herein, the amount of zeaxanthin was significantly lower. Despite employing the same extraction solvent, the strains were isolated from different environments, which clearly influenced their metabolomics.

Echinenone was not detected in the strain *Nodosilinea nodulosa* LEGE 06104 analyzed herein. Different results were obtained by Morone et al. [13], who reported 46.27 μg/g in an ethanol extract of the same species collected in a different location (*Nodosilinea nodulosa* LEGE 06102). Similarly, echinenone was reported in an acetone extract of a different species of the same genus (*Nodosilinea* (*Leptolyngbya*) *antarctica* LEGE13457) by Lopes et al. [17], appearing in higher amounts than that obtained herein (6.48 mg/g). The presence of echinenone constitutes an added value for a natural extract, since this compound is described as an essential carotenoid involved in photoprotection thanks to the presence of a carbonyl group, which seems to be mostly responsible for this bioactivity [18].

After analyzing the carotenoid production patterns of the genera *Phormidium*, *Oscillatoria*, and *Leptolyngbya*, Palinska et al. [19] concluded that, in general, strains from the same genera presented identical pigment patterns, with β-carotene and zeaxanthin being the most common. Looking at the qualitative profile of the strains analyzed herein, it can also be noticed that cyanobacteria present a very similar pigment pattern, with zeaxanthin, chlorophyll-*a* and β-carotene standing out relative to the other identified compounds (Figure 2, Table 1).

#### 2.2.2. Phycobiliproteins (PBPs)

Thanks to their intense and attractive colors, PBPs have been used as natural dyes in foods, nutritional supplements, and cosmetics, as well as fluorescent markers in immunoassays [20]. Being water-soluble proteins, PBPs were quantified spectrophotometrically in aqueous extracts. *Leptolyngbya boryana* LEGE 15486 presented the highest content of phycocyanin (PC), followed by *Cephalothrix lacustris* LEGE 15493, *Leptothoe* sp. LEGE 11479 and *Nodosilinea nodulosa* LEGE 06104 (*p* < 0.05) (Table 2). Regarding Allophycocyanin (APC) content, the cyanobacteria extracts presented the same order (*p* < 0.05). In contrast, phycoerythrin (PE) occurred in larger quantities in *Leptothoe* sp. LEGE 11479 (*p* < 0.05).

The results obtained herein are in line with the expectations, since the aqueous extracts of the strains *Leptolyngbya boryana* LEGE 15486 and *Cephalothrix lacustris* LEGE 15493 present the most intense blue color, characteristic of PC. Accordingly, the aqueous extract of *Leptothoe* sp. LEGE 11479, presenting the most intense pink color, was the richest in PE (Table 2). These strains have already been analyzed for their content of PBPs by Favas et al. [21], our results being of the same order of magnitude as those previously obtained.

The exploitation of cyanobacteria as PBP producers has been the subject of several studies, with variable results throughout the literature. For instance, Schipper et al. (2020) evaluated the genus *Leptolyngbya* as a potential producer of PBPs, pointing out this genus as a possible alternative to *Arthrospira platensis* for the production of PC-rich biomass [22]. In a tentative experiment to optimize pigment production, the authors varied different parameters, concluding that increased temperatures promoted higher biomass and PC productivity. It is important to note that, based on phylogenetic, morphological and ecological data, it has recently been proposed to transfer *Arthrospira platensis* to the genus *Limnospira* [23]. A study by Basheva and co-workers [24] showed that two strains of *Leptolyngbya boryana* produced significant amounts of both PC and APC (0.264 and 0.171 mg/mL), demonstrating significant potential for using these strains in biotechnological production of PBPs. Interestingly, the authors clarified that *Leptolyngbya* strains that belong to the same species (*Leptoplyngbya boryana*) showed significant differences in the amount of PBPs produced; the qualitative pigment profiles of the two *Leptoplyngbya boryana* strains was similar, probably due to their common taxonomic position. The difference in the PBP contents of different strains belonging to the same species was thus a reason to conclude that the production of these pigments was strictly individual and most likely related to the characteristics of each cyanobacterial strain. The findings of the work undertaken by Basheva and co-workers [24] are in agreement with the results obtained herein for *Leptolyngbya boryana* LEGE 15486, which was the strain with the highest contents of PC and APC. The same work revealed that the studied *Nodosilinea* strains had similar amounts of pigments, with PE being dominant, which contrasts with our results, where PC was the major PBP of *Nodosilinea nodulosa* LEGE 06104 (Table 2). Regarding *Leptothoe* sp. LEGE 11479, higher amounts of PE, followed by PC and APC, have also been reported [12].

PBPs from cyanobacteria have great relevance for biotechnological applications, not only for their colors but also for the associated antioxidant, anticancer and anti-inflammatory capacities [23]. Although strain selection is the determinant for the production of these compounds, the whole bioprocess needs to be carefully optimized to achieve the highest yield, namely through optimization of cyanobacteria culture conditions, such as light, nitrogen and carbon sources, pH, temperature and salinity, and the selection of the best solvent for PBP extraction from the biomass.

### 2.3. Biological Activities

#### 2.3.1. Antioxidant Potential

The relevant physiological free radicals O_2_^•−^ and ^•^NO, among the most representative ROS and RNS, responsible for oxidative and nitrosative damage in the human body, were used to determine the antioxidant potential of cyanobacteria extracts.

##### Superoxide Anion Radical Scavenging

**Figure 3:**
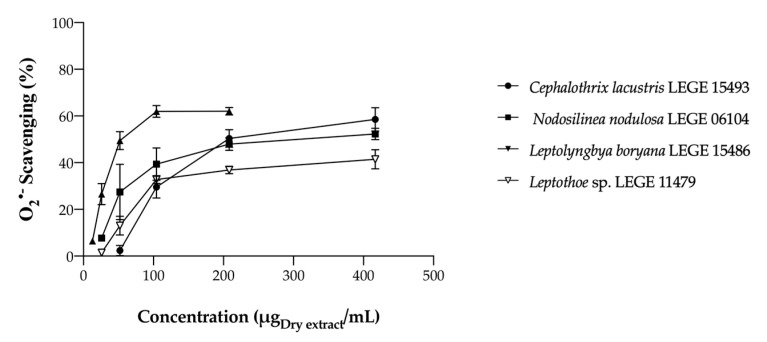
Superoxide anion radical (O_2_^•−^) scavenging activity of aqueous cyanobacteria extracts. Values are expressed as the mean ± SD of at least three independent experiments, performed in duplicate.

While the acetone extracts revealed weak activities, the aqueous ones were significantly more effective, only reaching IC_50_ values for O_2_^•−^ scavenging. The dose-dependent behavior of aqueous cyanobacteria extracts is displayed in Figure 3, while the calculated IC values are presented in Table 3.

*Leptolyngbya boryana* LEGE 15486 was the most effective strain, showing the lowest IC_50_ value (49.24 μg/mL), followed by *Cephalothrix lacustris* LEGE 15493 (178.06 μg/mL), and *Nodosilinea nodulosa* LEGE 06104 (233.56 μg/mL). *Leptothoe* sp. LEGE 11479 did not reach IC_50_ under the tested concentrations; however, for the highest concentration tested (417 µg/mL), O_2_^•−^ scavenging reached 41%. The antioxidant potential observed in the aqueous extracts may be attributed to their richness in PBPs. Indeed, a correlation can be established between O_2_^•−^ scavenging and PBP content and a negative, although non-significant correlation, was observed between PBP content and IC values, showing that these compounds contribute to the scavenging of this free radical (Table 2 and Table 3).

Although still scarce, some studies have reported on the radical scavenging ability of cyanobacteria extracts. Lopes et al. [17] performed the same assay using aqueous and acetone cyanobacteria extracts, and concluded that acetone extracts were more effective than ethanolic ones for O_2_^•−^ scavenging. In their work, *Nodosilinea* (*Leptolyngbya*) *antarctica* LEGE13457 acetone extract presented the lowest IC_50_ value (0.319 mg/mL). In contrast, the acetone extracts of our strains were unable to reach any IC value. Assuncão and co-workers [25] also evaluated the antioxidant potential of the marine cyanobacterium *Synechocystis salina,* exploring various solvents including acetone. The authors concluded that ethanol extract was the only one able to reach IC_50_ (1073.09  ±  17.38 µg/mL), unlike the acetone one, as in our work, allowing us to assume that more polar solvents are more effective in O_2_^•−^ scavenging. Accordingly, in another study conducted by Morone and co-workers [12], aqueous extracts were more effective and presented lower IC_50_ values than acetonic ones for O_2_^•−^ scavenging, corroborating our results.

##### Nitric Oxide Radical (^•^NO) Scavenging

**Figure 4:**
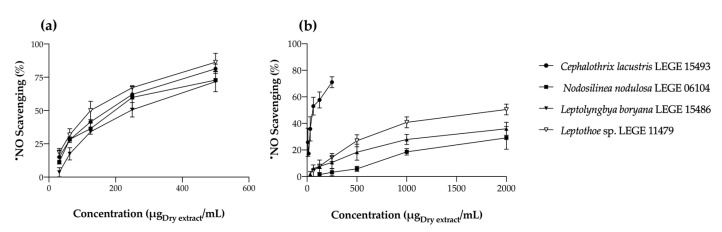
Nitric oxide radical (^•^NO) scavenging activity of acetonic (**a**) and aqueous (**b**) cyanobacteria extracts. Values are expressed as the mean ± SD of at least three independent experiments, performed in duplicate.

The ^•^NO scavenging capacities of the cyanobacteria extracts under study are displayed in Figure 4.

All extracts presented dose-dependent behavior (Figure 4). In general, acetonic extracts were more effective in ^•^NO scavenging than aqueous ones, with all of them reaching IC_50_ (Table 4). Among the acetonic extracts, that of *Leptothoe* sp. LEGE 11479 was the most effective, presenting the lowest IC_50_ value (129.47 μg/mL), being closely followed by *Nodosilinea nodulosa* LEGE 06104 (186.50 μg/mL) (*p* > 0.05). The aqueous extract of *Cephalothrix lacustris* LEGE 15493 seems to be an exception, as it was the one with the best ability to scavenge ^•^NO (IC_50_ = 36.68 μg/mL) (Table 4).

According to statistical analysis, it seems that in acetonic extracts, compounds 3, 7, and 16 contribute to ^•^NO scavenging, a negative correlation being observed (*p* < 0.01) between the contents of these carotenoids and the calculated IC values. On the other hand, compounds 11 and 17 presented a positive correlation with ^•^NO scavenging (*p* < 0.01). According to the literature, most carotenoids have been described as having a high ability to scavenge free radicals; however, when considering a complex extract, synergic and antagonist effects have to be considered, depending on the joint action of all the compounds.

As with O_2_^•−^, there is a lack of knowledge on the ^•^NO scavenging ability of cyanobacteria extracts, with few reports found in the literature. In a recent study, Assunção and co-workers [26] explored the biotechnological potential of a strain of the genus *Chroococcidiopsis* through several series of successive extractions. The authors reported the best radical scavenging with PBS (IC_50_ = 199.60 μg/mL) and water (IC_50_ = 216.20 μg/mL). In contrast to our results, the acetone extract explored by the authors did not reach IC_50_. Another study by the same research group [25], exploring the marine cyanobacterium *Synechocystis salina*, reported an IC_50_ value of 727.94 µg/mL for an acetonic extract for ^•^NO scavenging. The IC values obtained by us were significantly better, demonstrating the potential of the strains explored herein in the scavenging of nitrosative free radicals.

#### 2.3.2. Anti-Inflammatory Potential

##### Lipoxygenase Inhibition

**Figure 5:**
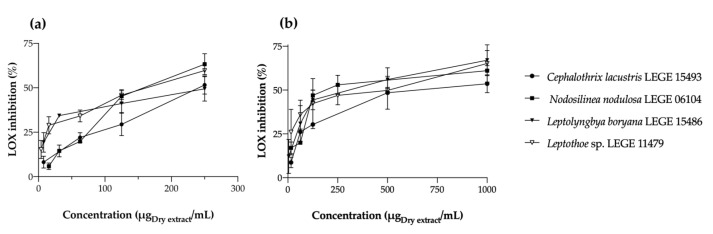
Lipoxygenase (LOX) inhibition by acetonic (**a**) and aqueous (**b**) cyanobacteria extracts. The percentage of LOX inhibition is expressed as mean ± SD of at least three independent experiments, performed in duplicate.

Lipoxygenases (LOXs) comprise a varied class of oxidative enzymes known to play an important role in inflammatory responses. These dioxygenases are involved in the regulation of inflammatory responses by generating pro-inflammatory mediators, known as leukotrienes, or anti-inflammatory mediators, known as lipoxins. LOX enzymes act by catalyzing the insertion of O_2_ into poly-unsaturated fatty acids (PUFAs), such as arachidonic and linoleic acid, generating oxidized phospholipids that induce alternative signalling pathways and additionally affect the biophysical properties of cellular membranes [27]. Cyanobacteria extracts were thus analyzed for their ability to inhibit LOX activity, and the results of their dose-dependent behavior is displayed in Figure 5.

With the exception of *Leptolyngbya boryana* LEGE 15486, whose aqueous extract presented a better IC_50_ than the acetonic one (142.75 vs. 196.37 μg/mL, respectively), all acetone extracts had lower IC_50_ values, proving to be more effective at inhibiting LOX (Table 5). Among the acetone extracts, *Nodosilinea nodulosa* LEGE 06104 stood out for having the lowest IC_50_ value (94.81 μg/mL, *p* < 0.05), while *Cephalothrix lacustris* LEGE 15493 was the least effective (251.69 μg/mL). According to the statistical analysis, there is a negative correlation (*p* < 0.01) between compounds **2**, **3**, **5**, **14**, and **18** and the calculated IC values, suggesting the contribution of these compounds to LOX inhibition. For instance, β-carotene (**18**) has been previously reported to be an efficient LOX inhibitor, directly influencing the enzyme through a non-competitive inhibition mechanism. This compound keeps LOX in its reduced form of Fe(II), and the oxidation of linoleic acid is prevented [28]. On the other hand, it seems that the presence of chlorophyll-*a* (**6**) prevents LOX inhibition, once a positive correlation is verified (0.817, *p* < 0.05).

The studies exploring cyanobacteria extracts for LOX inhibition are scarce. Jensen et al. [29] explored an *Arthrospira platensis* (currently *Limnospira platensis* (Gomont) K. R. S. Santos and G. S. Hentschke comb. nov.)-based aqueous cyanophyta extract in LOX inhibition and concluded that the inhibition of its enzymatic activity was specifically associated with the non-PC fraction, suggesting that, beyond PBPs, other compounds present in aqueous extracts may contribute to LOX inhibition. Fagundes et al. [30] explored a phytosterol-rich extract of *Phormidium autumnale* for LOX inhibition and obtained an IC_50_ of 58.20 μg/mL, which is lower than the values obtained herein for acetone extracts, but with a similar order of magnitude. As with carotenoids, phytosterols are low polar compounds that may also be present in acetone extracts and, together with carotenoids, contribute to the biological activities observed herein. Conversely, there are significantly more reports focusing on cyclooxygenase (COX), an enzyme that, along with LOX, is involved in the biosynthesis of eicosanoids (prostaglandins, tromboxanes and leukotrienes), using the arachidonic acid released from membrane phospholipids as substrate. For instance, Da Costa and co-workers [31] demonstrated that a lipidic extract of *Gloeothece* sp., mainly composed of glycolipids and phospholipids, was capable of inhibiting COX by 58% at a concentration of 10 μg/mL. Although the results of our study demonstrated that acetone extracts were more promising than aqueous ones, numerous studies report PBPs being associated with the ability to inhibit pro-inflammatory enzymes. Pagels and co-workers [32] found that a PBP-rich extract of *Cyanobium* sp. was capable of reducing COX-1 and COX-2 activity in a dosage of 100 μg/mL. Similarly, Reddy and his team [33] observed that PC from *Spirulina platensis* also reduced COX-2 activity in a dosage between 1-30 μg/mL. Altogether, the present results demonstrate that acetonic extracts are more promising than aqueous ones regarding LOX inhibition, thus being worthy of further exploitation.

##### Effect in LPS-Stimulated RAW 264.7 Cells

**Figure 6:**
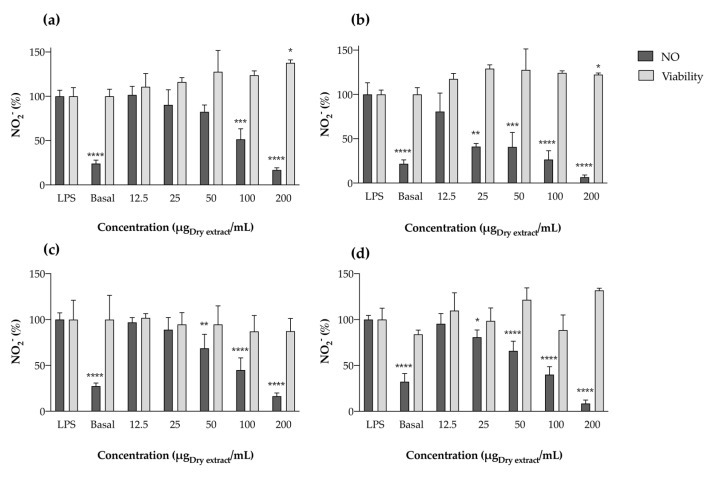
Nitric oxide production by RAW 264.7 cells in the presence of cyanobacteria acetone extracts: *Cephalothrix lacustris* LEGE 15493 (**a**), *Nodosilinea nodulosa* LEGE 06104 (**b**), *Leptolyngbya boryana* LEGE 15486 (**c**), and *Leptothoe* sp. LEGE 11479 (**d**), after stimulation with lipopolysaccharide (LPS). Results are expressed as % of nitrite (NO_2_^−^) relative to the control stimulated with LPS. “Basal” represents the nitric oxide produced by RAW 264.7 cells without LPS stimulation. Results are expressed as the mean ± SD of at least four independent assays, performed in duplicate. * *p* < 0.05, ** *p* < 0.01, *** *p* < 0.001, **** *p* < 0.0001 (ANOVA, Tukey HSD).

The anti-inflammatory potential of cyanobacterial extracts was also explored in a cell system by determining their capacity to reduce ^•^NO in the macrophage cell line RAW 264.7 under LPS stimulation. A simple cell exposure to acetone extracts, without LPS stimulation, revealed the extracts did not affect basal NO levels in the range of concentrations tested, thus excluding possible pro-inflammatory action. When RAW 264.7 cells were pre-exposed to serial dilutions of the acetonic extracts and then stimulated with LPS during 22 h, a clear reduction in NO levels in the culture medium was observed, with no toxicity against macrophages under the tested concentrations (Figure 6). As for LOX inhibition, the strain *Nodosilinea nodulosa* LEGE 06104 exhibited the lowest IC_50_ value (41.45 ± 29.56 μg/mL), displaying the best ability to reduce NO and, consequently, the best anti-inflammatory potential. This extract was closely followed by *Leptothoe* sp. LEGE 11479, with no significant differences between the calculated IC_50_ values (77.39 ± 19.20 μg/mL). *Leptolyngbya boryana* LEGE 15486 and *Cephalothrix lacustris* LEGE 15493 ranked last, with IC_50_ values of 100.84 ± 16.29 and 107.90 ± 23.65 μg/mL, respectively. At the highest concentrations tested (200 μg/mL), *Nodosilinea nodulosa* LEGE 06104 presented a maximum percentage of NO reduction of 94%, closely followed by *Leptothoe* sp. LEGE 11479 (92%) and then by *Leptolyngbya boryana* LEGE 15486 and *Cephalothrix lacustris* LEGE 15493, with the same percentage of 84%.

A study by Gomes and co-workers [34] explored the anti-inflammatory potential of fractions of different polarities obtained from different cyanobacteria strains, using a similar methodology of LPS-stimulated RAW 264.7 macrophages. Two *Leptolyngbya* sp. (*Leptolyngbya* sp. LEGE 07075 and *Leptolyngbya* sp. LEGE 07084), which share the same genus as one of the strains explored herein, were part of the study. However, the authors did not report promising anti-inflammatory potential concerning these two strains. This is not in accordance with our study, since *Leptolyngbya boryana* LEGE 15486 demonstrated clear anti-inflammatory potential, with a significant reduction in NO in a dose-dependent manner (Figure 5). In a study conducted by Lopes and co-workers [17], acetonic extracts of four cyanobacteria were explored for their anti-inflammatory potential using the same cell model. The authors tested two species of the same genus as those evaluated herein (*Leptolyngbya* and *Nodosilinea*), and verified NO reduction in the RAW 264.7 culture medium; however, only the IC_25_ value was reached. Briefly, *Nodosilinea antarctica* LEGE 13457 presented an IC_25_ value of 22.2 μg/mL, while *Leptolyngbya*-like sp. LEGE 13412 presented an IC_25_ of 84.1 μg/mL. This highlights the potential of carotenoid-rich extracts of species of the genus *Nodosilinea* and *Leptolyngbya* for the exploitation of anti-inflammatory metabolites, and demonstrates that the strains evaluated herein are more promising than those previously reported. According to statistical analysis, in addition to compounds **5** and **14**, β-carotene (compound **18**) was also negatively correlated with the calculated IC values (−0.757, *p* < 0.05), pointing out this compound as a contributor to the bioactivity observed. In fact, it has already been reported that β-carotene suppresses NF-κB activation and iNOS expression in RAW 264.7 cells stimulated with LPS [35]. Beyond pigment-rich extracts, some isolated compounds have been explored using the same cell model. For instance, Okai and Higashi-Okay [36] have reported that pheophytin-*a* from *Enteromorpha prolifera* suppressed the production of O_2_^•−^, an important inflammatory mediator, in mouse macrophages. Soontornchaiboon et al. [37] explored violaxanthin from *Chlorella ellipsoidea* and verified that this xanthophyll inhibited the production of NO and prostaglandins by RAW 264.7 cells, through the downregulation of NF-kB.

**Figure 7:**
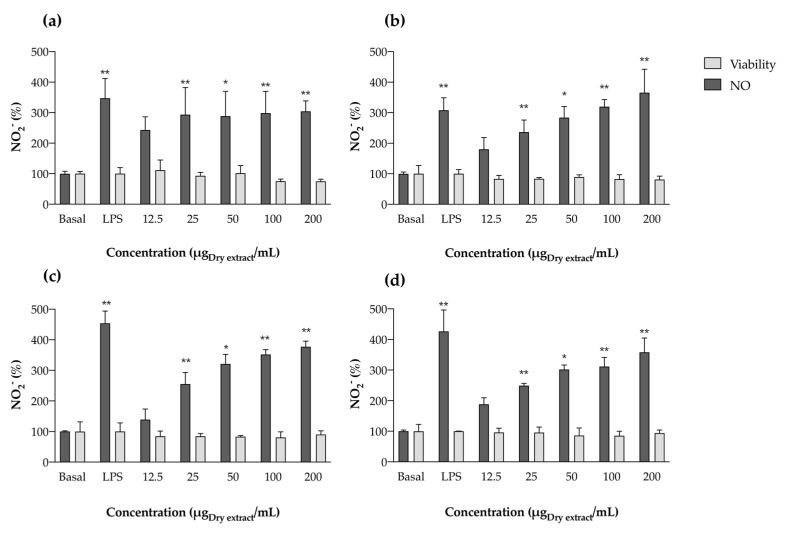
Nitric oxide production by RAW 264.7 cells in the presence of aqueous cyanobacteria extracts: *Cephalothrix lacustris* LEGE 15493 (**a**), *Nodosilinea nodulosa* LEGE 06104 (**b**), *Leptolyngbya boryana* LEGE 15486 (**c**), and *Leptothoe* sp. LEGE 11479 (**d**), without lipopolysaccharide (LPS) stimulation. Results are expressed as % of nitrite (NO_2_^−^) relative to the basal values produced by untreated cells (Basal). Results are expressed as the mean ± SD of at least four independent assays, performed in duplicate. * *p* < 0.05, ** *p* < 0.01 (ANOVA, Tukey HSD).

Concerning aqueous extracts, we noticed opposite behavior when compared with acetonic ones. While acetone extracts demonstrated anti-inflammatory potential by reducing NO levels, aqueous extracts showed pro-inflammatory behavior, by increasing the levels of this inflammatory mediator to a similar extent as LPS (Figure 7).

As for acetonic extracts, a first screening was performed by exposing RAW 264.7 cells to the aqueous extracts alone. The extracts did not present toxicity to the cells at the tested concentrations (Figure 7). However, after an incubation period of 24 h, NO produced by macrophages significantly increased, in some cases to values exceeding the bacterial LPS stimulation (Figure 7b), thus demonstrating pro-inflammatory behavior in aqueous extracts. The increase in NO values when compared with the untreated control (basal NO production) was evident for all strains under study, generally occurring in a dose-dependent manner (Figure 7b–d). Given the anti-inflammatory activity of PBPs already documented [7], and knowing that these molecules comprise the majority of the aqueous extracts prepared herein, we presuppose that other molecules able to be extracted with water, and with a similar mechanism of action to LPS, are responsible for these observations. In fact, this increase in NO production may be a result of the presence of cyanobacterial endotoxins (LPS), since in Gram-negative microorganisms, LPS are important outer membrane components [38]. These molecules are more prone to extraction with more polar solvents, such as water, causing macrophage activation through interactions with TLR4 present in cell membranes [39]. Lopes and co-workers [17] had previously reported that all ethanol (70%) extracts increased the levels of NO produced by RAW 264.7 on their own, suggesting pro-inflammatory potential, and corroborating the results obtained herein with water. In this regard, in order to evaluate the anti-inflammatory potential of PBP-rich extracts, further purification steps should follow.

**Figure 8:**
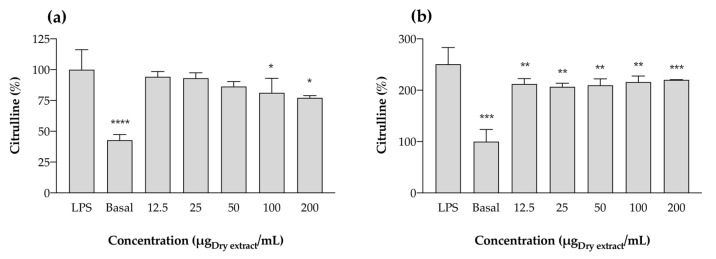
Citrulline production by RAW 264.7 cells in the presence of acetonic (**a**) and aqueous (**b**) extracts of *Cephalothrix lacustris* LEGE 15493. For the acetonic extract (**a**), results are expressed as % of citrulline relative to the control (Basal) after stimulation with LPS. For the aqueous extract (**b**), results are expressed as % of citrulline relative to the control (Basal) without LPS stimulation. “Basal” represents the citrulline production by untreated cells without LPS stimulation. Results are expressed as the mean ± SD of at least four independent assays, performed in duplicate. * *p* < 0.05, ** *p* < 0.01, *** *p* < 0.001, **** *p* < 0.0001 (ANOVA, Tukey HSD).

To shed some light on the mechanisms of anti-inflammatory and pro-inflammatory activity observed for acetonic and aqueous cyanobacteria extracts, respectively, a further assay was undertaken. L-Citrulline, an amino acid of the urea cycle that results from the activity of iNOS and is produced in stoichiometric amounts with NO, was measured. Given the chemical similarity and behavior of the aqueous and acetonic extracts, only one extract of each kind was selected to predict the mechanism of action. For this purpose, *Cephalothrix lacustris* LEGE 15493, the sample available in a higher amount, was screened, and the results are displayed in Figure 8.

For acetone extracts, a reduction in the percentage of citrulline was observed when compared with the LPS-stimulated control, which means that the extracts are directly interacting with iNOS. In this regard, the reduction in NO levels previously observed (Figure 6) occurs through at least two different mechanisms: NO scavenging (Figure 4, Table 4), and interference with iNOS expression (Figure 8). Given these results, it is demonstrated that acetone cyanobacteria extracts have anti-inflammatory activity. Conversely, in the case of aqueous extracts, there is pro-inflammatory activity. Once citrulline is produced by the metabolization of L-arginine through the action of iNOS, an increment in the concentration of this amino acid also requires an increment in the expression of iNOS. Once the overexpression of iNOS occurs through the activation of TLR4, we can deduce that the aqueous extracts may contain cyanobacteria endotoxins that produce a pro-inflammatory response in a similar way as the bacterial LPS.

Apart from pigments, cyanobacteria are known for the production of different molecules with anti-inflammatory activity, acting via different mediators and enzymes. Among others, compounds belonging to the classes of peptides, polysaccharides and lipids have been reported to reduce iNOS and COX mRNA expression levels, downregulate inflammatory cytokines like IL-1, IL-2, IL-6, IL-8, TNF-α and NF-kB, and reduce the formation of free radicals involved in the inflammatory process, among other mechanisms [7,40,41] Altogether, the results of the present work prove the importance of cyanobacteria as a sustainable resource in the search for new anti-inflammatory compounds.

## 3. Materials and Methods

### 3.1. Cyanobacteria Strains

In this project, the anti-inflammatory potential of cyanobacteria was assessed by exploring acetone and aqueous extracts of four filamentous strains: *Cephalothrix lacustris* LEGE 15493, *Leptolyngbya boryana* LEGE 15486, *Nodosilinea nodulosa* LEGE 06104 and *Leptothoe* sp. LEGE 11479 (Figure 1). *Cephalothrix lacustris* LEGE 15493 and *Leptolyngbya boryana* LEGE 15486 were isolated from a Portuguese marine environment, while *Nodosilinea nodulosa* LEGE 06104 and *Leptothoe* sp. LEGE 11479 were isolated from Brazilian freshwater. All the strains were maintained in the Blue Biotechnology and Ecotoxicology Culture Collection (LEGE CC) at the Interdisciplinary Centre of Marine and Environmental Research (CIIMAR). These strains were selected for their capacity to produce secondary metabolites with interesting biological activities, and for their low toxicity towards mammal cells, already reported in previous work undertaken by our research group [21].

#### Identification of the Strains

The identification of the studied strains was performed based on morphological and 16S rRNA gene phylogenetic analysis. For morphological analysis, the strains were observed and photographed under a Leica DMLB microscope (Leica Microsystems, GmbH, Wetzlar, Germany). To determine the phylogenetic position of our strains among other Cyanobacteria, we aligned their 16S rRNA nucleic acid sequences with reference strains of Leptolyngbyalaes, Nodosilineales, Oscillatoriales and Pseudanabaenales. The sequences were aligned using ClustalW in MEGA11: Molecular Evolutionary Genetics Analysis version 11 [42], and the final dataset contained 124 sequences with 955 informative sites. The phylogenetic trees were built using Maximum Likelihood analysis. GTR+G+I evolutionary model was selected by MEGA 11. The robustness of the ML tree was estimated by bootstrap percentages using 1000 replications, using IQ-Tree online version v1.6.12 [43].

### 3.2. Culture Conditions and Biomass Collection

A scale-up culture scheme was followed for cyanobacteria biomass production. Marine strains (*Leptothoe* sp. LEGE 11479 and *Nodosilinea nodulosa* LEGE 06104) were cultured in Z8 medium, with 25 g/L of marine salt and 1 mL/L of vitamin B12, while freshwater strains (*Leptolyngbya boryana* LEGE 15486 and *Cephalothrix lacustris* LEGE 15493) were grown in Z8 medium [44]. The four strains were cultured in the bioterium of aquatic organisms (BOGA) at CIIMAR and maintained at 25 °C under a light/dark cycle of 16 h/8 h respectively, with a light intensity of 10–30 μmol photons m^−2^ s^−1^. The initial cultivation volume was 40 mL, followed by a 10-fold scale-up to 400 mL and 4 L. The scale-up steps were made under aseptic conditions by collecting conglomerates of the grown cyanobacteria and transferring them into new flasks. A steady airflow input was introduced at the 4 L stage, providing natural aeration and improving growth. The cyanobacterial biomass was harvested by filtration with a net mesh (pore size 30 µm), commonly used for filamentous strains. Excess NaCl in the culture medium of the marine strains was removed by washing the filtrated biomass with distilled water. The fresh concentrated biomass was frozen and freeze-dried (LyoQuest, Telstar; Barcelona, Spain) under reduced pressure (0.1 mbar with the condenser at −47 °C) and kept at −20 °C until extract preparation.

### 3.3. Extracts Preparation

Acetone and aqueous extracts of each strain were sequentially prepared, aiming to extract compounds with different polarities, while making use of the same dry biomass. For the acetone extracts, 1g of dry biomass was suspended in 30 mL of 100% acetone in a glass Erlenmeyer flask and sonicated in an ultrasonic bath (Fisherbrand^®^-FB15053, Loughborough, UK) for 5 min. Temperature was kept under control to avoid degradation of thermolabile bioactive compounds. The mixture was filtered to allow separation between the acetone extract and the cell debris (pellet), which were re-extracted 4 times with the same solvent. The resulting filtrate was evaporated under reduced pressure (BUCHI R-210 Rotary Evaporator; 200 mBar), maintaining the water bath temperature below 35 °C. The pellet resulting from acetone extraction was left to dry in the fume hood overnight. Afterwards, it was re-extracted with 30 mL of distilled water, following a similar methodology to that mentioned above. After sonication, the mixture was transferred to a falcon tube and centrifuged at 10,000 Gs for 5 min at 4 °C (Thermo Scientific^TM^ HERAUS MegafugeTM 16R, USA). The supernatants were combined and the extraction process was repeated 4 times. The resulting water extract was frozen and lyophilized. Both acetone and water extracts were kept at −20 °C until further analysis.

### 3.4. Chemical Analysis

#### 3.4.1. Carotenoid and Chlorophyll Profiling by HPLC-PDA

The pigment profile of the acetone cyanobacteria extracts was determined using a Waters Alliance 2695 high-performance liquid chromatography (HPLC) system equipped with a photodiode array (PDA) detector (USA), following the methodology previously reported by Morone et al. [12]. Data were processed using Empower^®^ 2 chromatography software (Waters, NJ, USA), the spectra data from all peaks being collected in the range 250 to 750 nm. Compounds were tentatively identified by comparing their retention times and UV–Vis spectra with those of authentic standards. Carotenoid quantification was achieved by measuring the absorbance recorded in the chromatograms relative to external standards at 450 nm.

Lutein, chlorophyll-*a*, zeaxanthin, β-cryptoxanthin, echinenone and β-carotene (Extrasynthese, Genay, Fance; Sigma-Aldrich, St. Louise, MO, USA; DHI, Horsholm, Denmark) were quantified using authentic standards. Unidentified carotenoids were quantified as zeaxanthin, the most representative xanthophyll, and chlorophyll-*a* derivatives as chlorophyll-*a*, the major cyanobacterial chlorophyll. Calibration curves were performed with five different concentration standards, selected as representative of the range of compound concentrations in the samples (lutein: 0.78–12.5 µg/mL; chlorophyll-*a*: 0.31–5.00 µg/mL; β-cryptoxanthin: 1.56–25.00 µg/mL; echinenone: 1.56–25.00 µg/mL; β-carotene: 3.13–50.00 µg/mL). The calibration plots and *r*^2^ values for the analyzed carotenoids and chlorophyll-*a* are shown in Table 6, where “*y*” represents the area of the peak in the chromatogram, and “*x*” represents the analyte concentration.

#### 3.4.2. PBP Profiles

Being water-soluble proteins, PBPs were spectrophotometrically quantified in the aqueous extracts. Aqueous extracts were resuspended in water to a final concentration of 0.5 mg/mL. PBPs were determined by measuring the absorbance of the resulting solutions at different wavelengths (562, 615, and 652 nm), using a cell with a 1 cm optical path. The quantification was performed in duplicate and PBP amounts calculated as previously described [45,46]. The results were expressed in µg of the respective PBP per mg of dry extract.

### 3.5. Biological Activities

#### 3.5.1. Antioxidant Potential

The antioxidant potential of the cyanobacteria extracts was screened by measuring their ability to scavenge the relevant physiological free radicals O_2_^•−^ and ^•^NO generated in vitro.

##### O_2_^•−^ Scavenging

The O_2_^•−^ scavenging activity of the extracts was determined following a methodology previously described [12]. Aqueous extracts were resuspended in water, while acetone ones were resuspended in DMSO. Serial dilutions of each extract were prepared in a phosphate buffer (19 µM, pH 7.4). The radical scavenging capacity of the samples was determined by monitoring their effect on the reduction in NBT induced by O_2_^•−^ with a Synergy HT Multidetection Microplate Reader operated by GEN5TM (Biotek, Bad Friedrichshall, Germany), in kinetic function at room temperature for 2 min at 562 nm. Gallic acid was used as positive control. The results were expressed as the percentage of radical scavenged in comparison to the untreated control. The corresponding dose–response curves were calculated with Graphpad Prism^®^ software (Version 9.2.0 for Windows).

##### ^•^NO Scavenging

^•^NO generated from sodium nitroprusside (SNP) was measured according to the method described by Lopes and co-workers [47]. As before, aqueous extracts were resuspended in water, while acetone ones were resuspended in DMSO. Serial dilutions of each extract were prepared in a phosphate buffer (100 mM, pH 7.4). After the incubation period, the absorbance of the chromophore formed by the chemical reaction was read at 562 nm using a Synergy HT Multidetection Microplate Reader operated by GEN5TM (Biotek, Bad Friedrichshall, Germany). Blanks were performed for each concentration. The percentage of free radical scavenged was calculated relative to the untreated control. Gallic acid was used as positive control.

#### 3.5.2. Anti-Inflammatory Potential

##### Enzymatic Assays

(a) LOX Inhibition

To study the inhibitory effect of cyanobacteria extracts in LOX, we followed a procedure previously described by Fernandes and co-workers [48], with minor modifications. Serial dilutions of the extracts under study were pre-incubated in a reaction mixture containing 20 µL of extract, 200 µL of phosphate buffer (pH 9.0) and 20 µL of soybean lipoxygenase (100 U/20 µL), for 5 min at room temperature. After the incubation period, 20 µL of substrate (linoleic acid, 4.18 mM in ethanol) was added to start the reaction. Absorbance was measured continuously at 234 nm with a Synergy™ HT plate reader (Biotek Instruments; Winooski, VT, USA) operated via Gen5 Software (version 2.0) over 3 min. LOX inhibition was calculated by comparing the rate of reaction of the extracts relative to the untreated control. Quercetin was used as positive control.

##### Cell Assays

The murine macrophage cell line RAW 264.7 was selected as a model for prediction of the anti-inflammatory potential of the cyanobacteria extracts under study. Knowing that iNOS inhibitors represent an important therapeutic advance in the management of inflammatory diseases, the anti-inflammatory potential of cyanobacteria extracts was explored in this cell model after induction with LPS. The capacity of cyanobacteria extracts to modulate the activity of iNOS was evaluated by measuring the levels of ^•^NO and L-citrulline, both derived from enzymatic conversion of the amino acid L-arginine. The cytotoxicity of the extracts was also evaluated by measuring the cell dehydrogenases’ activity through the MTT assay in order to establish a range of non-toxic concentrations.

(a) Cell Maintenance

The murine macrophage cell line RAW 264.7 was maintained in Dulbecco’s Modified Eagle medium (DMEM), supplemented with GlutaMAX™-I, 10% of inactivated FBS, 100 U/L penicillin and 100 µg/mL streptomycin, in a humidified atmosphere of 5% CO_2_ at 37 °C. Cells were maintained in ventilated culture flasks, and the culture medium was renewed every two days. After reaching 80–90% confluence, cells were detached using a cell scraper in order to proceed with their seeding or transference. The assays were performed with RAW 264.7 cell passages between 6 and 18.

(b) Toxicity to RAW 264.7 Cells

The cytotoxicity of the extracts was monitored through the 3-(4,5-dimethylthiazole-2-yl)-2,5-diphenyltetrazolium bromide (MTT) assay, as previously described [49]. The absorbance of the solubilized formazan salts was quantified spectrophotometrically at 515 nm using a Synergy HT Multi-Detection microplate reader (Biotek, Germany) operated via GEN5 software. Cytotoxicity was expressed as the percentage of cell viability relative to the untreated control (0.5% DMSO).

(c) NO Release by RAW 264.7 Cells

The effect of cyanobacteria extracts on ^•^NO produced by LPS-stimulated macrophages was evaluated by measuring the accumulated nitrite (NO_2_^−^) in the extracellular medium as an indicator of ^•^NO production, following a previously established protocol [49]. The absorbance of the formed chromophore was read at 562 nm using a Synergy HT Multi-Detection microplate reader (Biotek, Germany) operated via GEN5 software. The production of ^•^NO was also measured in the presence of the extracts without LPS stimulation in order to infer the effect of the extracts themselves in the basal NO production.

(d) Determination of L-Citrulline Levels

The levels of L-citrulline in the supernatant of LPS-stimulated RAW 264.7 cells were determined according to the procedure described by Barbosa and co-workers [50]. Due to the similar behavior among acetone and aqueous extracts, only one of each kind was chosen to perform the citrulline assay, taken as a mechanistic study. The effect of the extracts on L-citrulline levels was calculated taking the LPS-stimulated control as reference for the acetone extracts, and the non-stimulated control as reference for aqueous extracts. *Cephalothrix lacustris* LEGE 15493, the sample available in a higher amount, was selected to perform this mechanistic study.

### 3.6. Statistical Analysis

Statistical analysis was performed using IBM SPSS STATISTICS software, version 27.01.0, IBM Corporation, New York, NY, USA (2020). Data were analyzed for normality and homogeneity of variances using Kolmogorov–Smirnov and Leven’s tests, and then submitted to one-way ANOVA using a Tukey’s HSD (honest significant difference) as a post hoc test, or to a *t*-test. A Pearson correlation test was used to compare normalized expression data between the chemical profile and the biological activities of both aqueous and acetonic cyanobacteria extracts. The results for the calculated IC values were expressed as means ± SD (μg/mL) of at least three independent assays, performed in duplicate, for free radicals and enzymatic analysis, and at least five independent assays performed in duplicate, for cell analysis.

## 4. Conclusions

In the present work, pigment-rich extracts of cyanobacteria biomass proved promising for pharmacological and biotechnological applications as anti-inflammatories, in a biorefinery approach where a single biomass is subjected to sequential extraction steps. The non-cytotoxic acetone and aqueous extracts of the four strains exhibited promising results by interfering with different mediators and enzymes with key importance in the development of inflammation. Regarding free radicals, all the extracts demonstrated encouraging results, with aqueous ones being more effective at scavenging free radicals of oxygen (O_2_^•−^) and acetonic ones at scavenging free radicals of nitrogen (^•^NO), with species from the genus *Leptolyngbya* standing out. Acetone extracts also demonstrated anti-inflammatory capacity in the enzymatic and cell systems studied, with *Nodosilinea nodulosa* LEGE 06104 and *Leptothoe* sp. LEGE 11479 being the strains with greater potential. Although it was verified by the present results that acetone cyanobacteria extracts modulate iNOS expression, more studies are worthy of being conducted to fully understand the mechanisms of action and potential side effects of cyanobacteria’s bioactive metabolites. However, the current findings highlight these valuable resources for the development of anti-inflammatory therapies, providing innovative multitarget solutions in the field of inflammation.

## Figures and Tables

**Table 1:** Carotenoid and chlorophyll contents (mg/g _dry extract_) in acetone cyanobacteria extracts, determined by HPLC-PDA ^1,2^.

Peak	Compound	RT (min)	*Cephalothrix lacustris* LEGE 15493	*Nodosilinea nodulosa* LEGE 06104	*Leptolyngbya boryana* LEGE 15486	*Leptothoe* sp. LEGE 11479
**1**	Chlorophyll-*a* derivative	9.7	nd	nd	nq	2.177 ± 0.066
**2**	Chlorophyll-*a* derivative	10.1	0.018 ± 0.004 ^c^	1.269 ± 0.179 ^a^	nd	0.574 ± 0.059 ^b^
**3**	Chlorophyll-*a* derivative	11.0	nd	nd	nd	0.321 ± 0.028
**4**	Lutein	12.5	0.578 ± 0.033 ^c^	1.044 ± 0.065 ^b^	0.381 ± 0.018 ^c^	1.383 ± 0.097 ^a^
**5**	Unidentified Carotenoid	13.6	1.368 ± 0.060 ^c^	11.996 ± 0.037 ^a^	2.321 ± 0.013 ^b^	2.099 ± 0.103 ^b^
**6**	Chlorophyll-*a*	14.9	17.461 ± 0.186 ^a^	5.031 ± 0.162 ^c^	10.949 ± 0.604 ^b^	14.740 ± 1.533 ^a^
**7**	Zeaxanthin	15.2	99.553 ± 0.515 ^c^	123.911 ± 0.865 ^b^	52.680 ± 0.399 ^d^	192.908 ± 3.198 ^a^
**8**	Chlorophyll-*a* derivative	16.6	0.532 ± 0.039 ^c^	1.934 ± 0.050 ^b^	0.512 ± 0.047 ^c^	3.663 ± 0.333 ^a^
**9**	Unidentified Carotenoid	17.4	nd	nd	7.661 ± 0.128	nd
**10**	Unidentified Carotenoid	20	8.588 ± 0.050 ^b^	18.944 ± 0.111 ^a^	1.134 ± 0.030 ^d^	2.060 ± 0.082 ^c^
**11**	Unidentified Carotenoid	21.4	2.219 ± 0.050 ^b^	3.156 ± 0.012 ^a^	3.496 ± 0.174 ^a^	1.068 ± 0.011 ^c^
**12**	Unidentified Carotenoid	22.9	nd	5.834 ± <0.001 ^a^	nd	2.485 ± 0.087 ^b^
**13**	β-Cryptoxanthin	24.0	2.033 ± 0.037 ^b^	2.793 ± 0.188 ^a^	1.686 ± 0.017^b^	2.577 ± 0.085 ^a^
**14**	Unidentified Carotenoid	24.7	nd	nd	2.261 ± 0.069	nd
**15**	Echinenone	25.3	1.785 ± 0.005 ^a^	nd	1.699 ± 0.079 ^b^	nd
**16**	Unidentified Carotenoid	26.9	2.969 ± 0.006 ^b^	2.455 ± 0.136 ^b^	1.374 ± 0.127 ^c^	3.901 ± 0.177 ^a^
**17**	Unidentified Carotenoid	28.0	10.324 ± 0.087 ^b^	nd	22.332 ± 0.168 ^a^	nd
**18**	β-Carotene	32.8	18.393 ± 0.250 ^b^	44.315 ± 1.471 ^a^	20.408 ± 3.198 ^b^	39.791 ± 8.117 ^a^
**19**	β-Carotene derivative	34.0	6.167 ± 0.147 ^c^	13.747 ± 0.046 ^a^	3.870 ± 0.026 ^d^	12.358 ± 0.252 ^b^
	Total carotenoids		153.978 ± 1.391 ^b^	228.194 ± 2.929 ^a^	121.304 ± 4.447 ^b^	260.629 ± 12.205 ^a^
	Total chlorophylls		18.012 ± 0.229 ^a^	8.234 ± 0.390 ^b^	11.462 ± 0.651 ^b^	21.476 ± 2.019 ^a^

^1^ Values are expressed as mean ± SD of four determinations; ^2^ Different superscript letters in the same row denote statistical differences at *p* < 0.05 (ANOVA, Tukey HSD, *t*-test); nd: not detected; nq: not quantified.

**Table 2:** Phycobiliproteins content (μg/mg _dry extract_) in aqueous cyanobacteria extracts ^1^.

Strains	Phycobiliproteins		
	Phycocyanin	Allophycocyanin	Phycoerythrin
*Cephalothrix lacustris* LEGE 15493	108.27 ± 0.54 ^b^	31.98 ± 0.38 ^b^	16.52 ± 0.61 ^b^
*Nodosilinea nodulosa* LEGE 06104	30.83 ± 0.28 ^d^	4.96 ± 0.44 ^d^	4.93 ± 0.18 ^c^
*Leptolyngbya boryana* LEGE 15486	204.52 ± 0.14 ^a^	41.61 ± 0.22 ^a^	14.42 ± 0.39 ^b^
*Leptothoe* sp. LEGE 11479	54.02 ± 1.76 ^c^	22.04 ± 0.87 ^c^	78.49 ± 3.01 ^a^

^1^ Different superscript letters in the same column denote statistical differences at *p* < 0.05 (ANOVA, Tuckey HSD).

**Table 3:** Inhibitory concentration (IC) values (µg _dry extract_/mL) obtained for O_2_^•−^ scavenging by aqueous cyanobacteria extracts ^1^.

Strains	IC_25_	IC_50_
*Cephalothrix lacustris* LEGE 15493	87.55 ± 1.07 ^a,b^	178.06 ± 9.82
*Nodosilinea nodulosa* LEGE 06104	66.72 ± 29.60 ^a,b^	233.56 ± 176.09
*Leptolyngbya boryana* LEGE 15486	25.19 ± 3.06 ^a^	49.24 ± 4.45
*Leptothoe* sp. LEGE 11479	111.08 ± 30.43 ^b^	nd

^1^ Different superscript letters in the same column denote statistical differences at *p* < 0.05 (ANOVA, Tukey HSD).

**Table 4:** Inhibitory concentration (IC) values (µg _dry extract_/mL) obtained for ^●^NO scavenging by aqueous and acetonic cyanobacteria extracts ^1^.

Strains	Aqueous Extracts		Acetonic Extracts	
	IC_25_	IC_5O_	IC_25_	IC_5O_
*Cephalothrix lacustris* LEGE 15493	15.91 ± 10.36 ^a^	36.68 ± 4.57	57.56 ± 2.02 ^a^	329.627 ± 7.54 ^a,b^
*Nodosilinea nodulosa* LEGE 06104	1617.62 ± 186.65 ^c^	nd	75.16 ± 13.52 ^a,b^	186.50 ± 47.90 ^a,b^
*Leptolyngbya boryana* LEGE 15486	835.31 ± 286.74 ^b^	nd	95.19 ± 5.24 ^b^	232.57 ± 36.46 ^b^
*Leptothoe* sp. LEGE 11479	472.99 ± 104.64 ^a,b^	1526.9 *	46.63 ± 11.40 ^a^	129.47 ± 23.87 ^a^

* IC_50_ only reached in one determination. ^1^ Different superscript letters in the same column denote statistical differences at *p* < 0.05 (ANOVA, Tuckey HSD).

**Table 5:** Inhibitory concentration (IC) values (µg _dry extract_/mL) obtained for lipoxygenase (LOX) inhibition by aqueous and acetonic cyanobacteria extracts ^1^.

Strains	Aqueous Extracts		Acetonic Extracts	
	IC_25_	IC_5O_	IC_25_	IC_5O_
*Cephalothrix lacustris* LEGE 15493	87.54 ± 26.48	375.51 ± 32.97 ^b^	96.77 ± 44.96	251.69 ± 29.54 ^b^
*Nodosilinea nodulosa* LEGE 06104	37.43 ± 15.13	206.02 ± 70.10 ^a^	42.44 ± 22.75	94.81 ± 40.83 ^a^
*Leptolyngbya boryana* LEGE 15486	68.94 ± 23.10	142.75 ± 21.91 ^a^	34.36 ± 13.94	196.37 ± 0.21 ^b^
*Leptothoe* sp. LEGE 11479	46.98 ± 27.78	484.04 ± 27.44 ^b^	25.92 ± 3.52	176.00 ± 3.49 ^a,b^

^1^ Different superscript letters in the same column denote statistical differences at *p* < 0.05 (ANOVA, Tuckey HSD).

**Table 6:** Calibration curves of authentic standards used for quantification of different carotenoids and chlorophylls.

Standards	Calibration Curve	*r* ^2^
Lutein	y = 93629588x + 13548	0.9999
Chlorophyll-a	y = 83010783x + 144987	0.9985
Zeaxanthin	y = 80009404x + 19635	0.9944
β-Cryptoxanthin	y = 28522518x + 8558	0.9992
Echinenone	y = 87231499x + 43970	0.9996
β-Carotene	y = 29605275x + 36236	0.9982

## Data Availability

Data will be available upon request to the corresponding author.

## Supplementary Materials

The following supporting information can be downloaded at: https://www.mdpi.com/article/10.3390/md22030131/s1, Figure S1: Maximum Likelihood phylogeny of the studied strains and their closest related genera. The bootstrap support is shown at the nodes. The strains studied in this article are marked in bold.
